# Clinicopathological Features and Pathogenesis of Thymoma Complicated with Alopecia Areata: A Multicenter, Matched Case Analysis

**DOI:** 10.3390/cancers17162672

**Published:** 2025-08-16

**Authors:** Xin Du, Xuehan Gao, Jian Cui, Xintao Yu, Cheng Huang, Yeye Chen, Chao Guo, Ye Zhang, Chao Gao, Xiayao Diao, Lei Yu, Shanqing Li

**Affiliations:** 1 Department of Thoracic Surgery, Peking Union Medical College Hospital, Chinese Academy of Medical Science & Peking Union Medical College, Beijing 100730, China; b2023001200@pumc.edu.cn (X.D.); gaoxh1299@163.com (X.G.); chenyeye@pumch.cn (Y.C.); guochao@pumch.cn (C.G.); zhangye10667@pumch.cn (Y.Z.); pk2009gc@126.com (C.G.); diaoxiayao@pumch.cn (X.D.); 2 Department of Thoracic Surgery, Beijing Tongren Hospital, Capital Medical University, Beijing 100730, China; cuijian_1996@163.com (J.C.); 15110204836@163.com (X.Y.); huangcheng@pumch.cn (C.H.)

**Keywords:** thymoma, alopecia areata, paraneoplastic syndrome, CD8^+^ T lymphocyte

## Abstract

Background: Thymoma is a malignant tumor originating from the thymic epithelium and can be associated with over 100 paraneoplastic syndromes (PNSs). Due to the low incidence of thymoma and the relative rarity of alopecia areata (AA) as an associated autoimmune disease, patients with thymoma combined with AA are relatively uncommon in clinical practice. As a result, the clinicopathological features and pathogenesis of such patients have been rarely investigated. Methods: This study retrospectively analyzed the clinical records of thymoma patients who underwent surgical treatment at Peking Union Medical College Hospital and Beijing Tongren Hospital from August 2014 to July 2019, with a focus on the clinicopathological features of thymoma patients with AA. Propensity score matching (PSM) was employed to create a 1:5 matched comparison group with thymoma patients without AA. Results: A total of 428 thymoma patients were included, among which 9 had AA. Using PSM, we matched 45 control patients without AA based on age and gender. The analysis revealed that thymoma patients with AA had a significantly higher proportion of myasthenia gravis (MG) [100.00% (9/9) vs. 66.67% (30/45), *p* = 0.049], although there were no significant differences between the AChR antibodies, Titin antibodies, MG severity, and the incidence of postoperative myasthenic crisis. However, the proportion of thymoma patients with AA who also had other PNSs besides MG was significantly higher [88.89% (8/9) vs. 6.67% (3/45), *p* < 0.001]. Additionally, CD4^+^/CD8^+^ T-cell inversion in the serum was observed at a much higher rate in thymoma patients with AA [100.00% (9/9) vs. 24.44% (11/45), *p* < 0.001]. Conclusions: We hypothesize that the pathogenesis of thymoma with AA differs from that of thymoma with MG, though there may be a correlation. The etiology of thymoma with AA may be attributed to abnormal autoimmune CD8^+^ T lymphocytes produced by the thymoma, which can also lead to other cytotoxic T-cell-mediated autoimmune diseases.

## Simple Summary

Thymoma is a rare tumor in the chest that can sometimes be linked to other diseases caused by the immune system attacking the body, such as alopecia areata, which leads to patchy hair loss. Because both thymoma and alopecia areata are uncommon, their relationship has rarely been studied. In this research, we reviewed medical records from two hospitals across a period of 5 years to compare thymoma patients with and without alopecia areata. We found that patients with alopecia areata often had other immune-related diseases and a high level of a certain type of immune cell called CD8^+^ T lymphocyte. This suggests that these cells may play an important role in causing alopecia areata and other immune problems in thymoma patients. The findings of this study provide valuable insights for the treatment of and pathogenesis research on thymoma with alopecia areata.

## 1. Introduction

Thymoma is a malignant tumor originating from thymic epithelial cells, with an annual incidence of approximately 1.2–2.6 per million [1]. It is also the most common anterior mediastinal tumor, accounting for about 50% of all anterior mediastinal tumors [2]. However, the etiology and pathogenesis of thymoma remain poorly understood [2,3]. Approximately 30–50% of thymoma patients have paraneoplastic syndromes (PNSs) [3,4]. According to research, 123 kinds of PNS have been reported so far [5], among which myasthenia gravis (MG) is the most common, accounting for about 25–40% of thymoma patients [6].

Alopecia areata (AA) is a common chronic organ-specific autoimmune disease, with an incidence of approximately 2% in the general population [7,8]. It is characterized by smooth, patchy hair loss on the scalp, but can also occur in any area with hair growth. Severe AA can lead to total hair loss (alopecia totalis) or even the complete loss of all body hair (alopecia universalis) [9]. AA, as a relatively rare autoimmune disease associated with thymoma, was first reported by Muller and Rochester in 1963 [10]. However, due to the relatively low incidence of thymoma and the common coexistence of more severe PNSs like MG, rare comorbidities such as AA are often overlooked. Currently, there are only about 20 reported cases of thymoma complicated by AA in the international literature, most of which are individual case reports, lacking a systematic analysis of the clinicopathological features and pathogenesis of patients with thymoma and AA.

This study retrospectively analyzes thymoma cases from two centers, Peking Union Medical College Hospital and Beijing Tongren Hospital, to examine the clinicopathological features and immunological test results of patients with thymoma complicated by AA over the past 5 years. This study aims to summarize the clinical and immunological characteristics of these patients, explore their potential pathogenesis, and provide valuable insights for the treatment of and further research on thymoma patients with AA.

## 2. Materials and Methods

### 2.1. Data Sources and Diagnostic Criteria

This study is a multicenter retrospective study, with case data derived from the Thymic Disease Database of Peking Union Medical College Hospital and Beijing Tongren Hospital. This study includes patients who underwent surgical treatment for thymoma between 1 August 2014 and 31 July 2019. Inclusion criteria: (1) patients who underwent thymoma surgery at the two hospitals after first onset; (2) postoperative pathology confirmed the diagnosis of thymoma; (3) complete clinical examination and follow-up data available; and (4) postoperative follow-up duration greater than 5 years. Exclusion criteria: (1) patients with concurrent other malignant or borderline tumors; and (2) patients who failed to follow up or missed scheduled check-ups.

According to the latest 5th edition of *WHO Classification of Thoracic Tumors*, published in 2021, the pathological subtypes of thymoma are classified as Type A (including atypical subtype), Type AB, Type B1, Type B2, Type B3, micronodular thymoma with lymphoid stroma, metaplastic thymoma, and lipofibroadenoma [11]. Thymomas are staged according to the Masaoka–Koga system and TNM staging, which includes Stages I, IIa, IIb, III, IVa, and IVb [12]. The classification of MG is based on the clinical classification of the Myasthenia Gravis Foundation of America (MGFA), which includes Type I, Type II, Type III, Type IV, and Type V [13]. The diagnosis of AA is based on typical skin manifestations, with the exclusion of the effects of radiotherapy and chemotherapy. In some cases, a skin biopsy showing typical histological features, such as lymphocytic infiltration around the hair bulbs and papillae, further confirms the diagnosis [9].

MG-associated antibodies serve as important indicators for the diagnosis of MG. The AchR antibody is detected using a radioimmunoassay, with a result greater than 0.5 nmol/L considered positive. The Titin antibody is detected by Western blotting, and the results are interpreted based on the intensity of the antigen staining band as negative (no color), questionable (very weak staining), weakly positive (weak staining), positive (strong staining), and strongly positive (staining intensity equal to that of the control band). The quantification of CD4^+^ and CD8^+^ T lymphocytes in serum and tissue was performed using flow cytometry. The normal ratio of CD4^+^/CD8^+^ T lymphocytes is 1–2.16, and a ratio of less than 1 is defined as ratio inversion.

This retrospective study was reviewed and approved by the ethics committees of Peking Union Medical College Hospital and Beijing Tongren Hospital with a waiver of informed consent (ethical approval numbers: K4735 and TRECKY2021-175, respectively).

### 2.2. Follow-Up and Symptom Assessment

Postoperatively, patients received a follow-up appointment once every month for the first 3 months, every 3 months after 6 months, and every 6 months after 1 year. The follow-up was conducted through a combination of outpatient visits and telephone consultations, with the follow-up period extending until 31 July 2024. Patients underwent chest CT scans every 6 months postoperatively, and after 2 years, if no tumor progression was observed, the frequency was reduced to annual chest CT scans. The follow-up primarily focused on changes in symptoms related to postoperative AA and other PNSs, as well as tumor recurrence and survival status.

### 2.3. Statistical Analysis

A data statistical analysis was performed using SPSS 26.0 software. Quantitative data with a normal distribution were expressed as mean ± standard deviation, while non-normally distributed quantitative data were expressed as median (interquartile range, IQR). For comparisons of differences between groups, an independent sample’s *t*-test was used for continuous variables with a normal distribution, a non-parametric test was used for continuous variables with a non-normal distribution, and the chi-squared test or Fisher’s exact test was applied for categorical variables of two independent samples. Propensity score matching (PSM) was conducted with a 1:5 matching ratio for the control group, with a caliper value set at 0.02. Two-sided tests were applied, and a *p*-value < 0.05 was considered statistically significant.

## 3. Results

**Figure 1:**
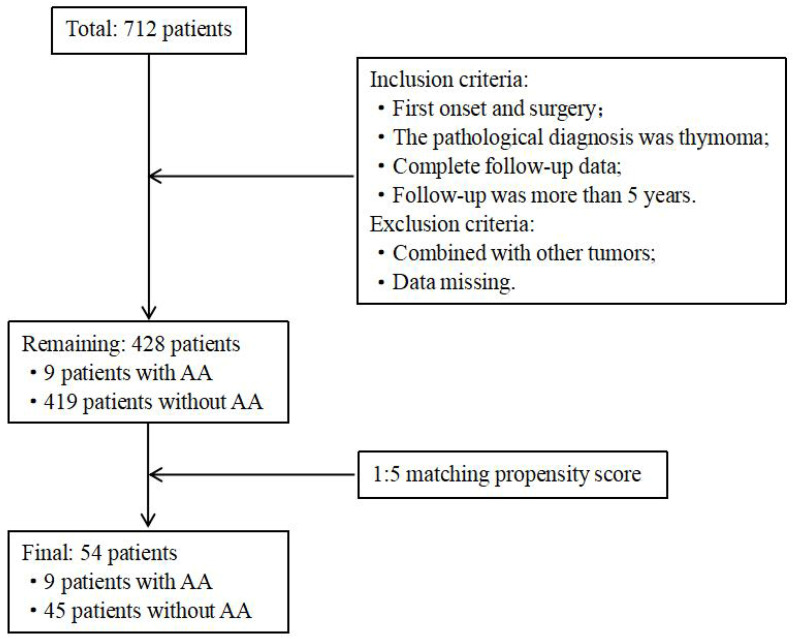
Flow chart for screening and matching study populations. AA, alopecia areata.

Figure 1 shows the flow chart of patient enrollment and matching screening. Between August 2014 and July 2019, a total of 712 thymoma surgeries were performed across two centers over a five-year period. According to the inclusion and exclusion criteria, a total of 428 thymoma patients were included, among which 9 patients (2.10%) were diagnosed with AA, and 419 patients (97.90%) did not have AA. Among the nine thymoma patients with AA, there were three males (1/3) and six females (2/3). The median age of onset for thymoma patients with AA was 46 (44, 53) years. Among these patients, there was one case of B1 thymoma (1/9) and eight cases of B2/B3 thymoma (8/9). Tumor staging revealed that one patient (1/9) was at Stage I, two patients (2/9) were at Stage III, and six patients (6/9) were at Stage IV. All patients were diagnosed with MG; one case (1/9) as Type III, one case (1/9) as Type IV, and four cases (4/9) as Type V of MG. Postoperatively, two patients (2/9) experienced myasthenic crises. All patients exhibited AchR antibodies in their serum, and seven patients (7/9) were also positive for Titin antibodies. All nine patients showed an inverted CD4^+^/CD8^+^ T lymphocyte ratio in their serum (Table 1).

To further verify whether the inversion of the CD4^+^ T/CD8^+^ T lymphocyte ratio in serum is associated with thymoma, we performed a flow cytometric analysis to assess the CD4^+^ T/CD8^+^ T lymphocyte ratios in both thymoma and peritumoral tissues. The results showed that in the thymoma patients with AA, the inversion of the CD4^+^ T/CD8^+^ T lymphocyte ratio was consistently observed in tumor tissues (9/9), aligning with the findings in the serum. However, no inversion of the CD4^+^ T/CD8^+^ T lymphocyte ratio was detected in peritumoral tissues (0/9), suggesting that the abnormal CD4^+^ T and CD8^+^ T lymphocyte ratios in serum may be associated with thymoma (Table 1).

The onset of AA in patients was consistently later than that of MG, with a median onset time of 7 (28.5) months after MG. Among the nine thymoma patients with AA, eight (8/9) were also diagnosed with other PNSs besides MG, including autoimmune myocarditis, autoimmune thyroiditis, dermatomyositis, leucoderma, Good’s syndrome, and more than 10 other PNSs. Postoperatively, AA symptoms improved in eight patients (8/9), with a median improvement time of 5 (5.25) months, which was later than the improvement time for MG symptoms. Three patients (3/9) experienced a recurrence of AA upon thymoma recurrence (Table 1).

To further study the clinicopathological and immunological characteristics of thymoma patients with AA, we conducted a comparative analysis with thymoma patients without AA. Due to the substantial difference in case numbers between the two groups, we used PSM to match for age and sex at a 1:5 ratio, resulting in a control group of 45 patients. Our analysis revealed that there was no significant difference in the proportion of pathological type B2/B3 between thymoma patients with AA and those without AA (8/9 vs. 25/45, *p* = 0.075). However, the proportion of patients who were at Stage IV was significantly higher in thymoma patients with AA compared to those without AA (6/9 vs. 4/45, *p* = 0.001) (Table 2).

Furthermore, all thymoma patients with AA also had MG, a difference compared to thymoma patients without AA (9/9 vs. 30/45, *p* = 0.049). However, there was no significant difference in the proportion of AchR and Titin antibody positivity in the serum between thymoma patients with AA and those without AA (9/9 vs. 38/45, *p* = 0.586 and 6/9 vs. 32/45, *p* > 0.999, respectively). Moreover, the proportion of MG Types IV–V among thymoma patients with AA was not significantly different from thymoma patients with MG but without AA (4/9 vs. 6/30, *p* = 0.197). Additionally, there was no significant difference in the proportion of postoperative myasthenic crises between the two groups (2/9 vs. 2/30, *p* = 0.223) (Table 2).

Regarding PNS associated with thymoma other than MG, thymoma patients with AA had a significantly higher prevalence compared to those without AA (8/9 vs. 3/45, *p* < 0.001). In addition, the inversion of the CD4^+^ T/CD8^+^ T cell ratio in the serum was significantly more frequent in thymoma patients with AA compared to those without AA (9/9 vs. 11/45, *p* < 0.001). However, no significant difference was observed between the two groups in the number of CD4^+^ T/CD8^+^ T lymphocytes inverted in tumor (9/9 vs. 39/45, *p*= 0.574) (Table 2).

## 4. Discussion

This study retrospectively analyzed the clinical records of thymoma patients over a five-year period from Peking Union Medical College Hospital and Beijing Tongren Hospital, revealing that the incidence of AA in thymoma patients was 2.10%, which is similar to the incidence rate in the general population. However, among thymoma patients with MG, the incidence was as high as 4.32%. Due to the relatively mild symptoms of AA, it is often overlooked in clinical practice, suggesting that the actual incidence of AA in thymoma patients may be significantly underestimated.

Following PSM for the nine thymoma patients with AA, an analysis revealed no significant differences in the serum levels of AchR and Titin antibodies among patients with MG between the two groups (*p* = 0.586 and *p* = 1.000). Additionally, there were no significant differences in the severity of MG (*p* = 0.197) or in the proportion of patients experiencing postoperative myasthenic crises (*p* = 0.223). These findings suggest that the pathogenesis of AA and MG in thymoma may be different. However, the proportion of MG occurrence was significantly higher in thymoma patients with AA compared to the thymoma patients without AA (*p* = 0.049). Thus, we hypothesize that while the pathogenesis of AA and MG in thymoma patients may differ, there may still be a relationship between the two. This is further supported by the fact that the onset and recovery of AA symptoms in thymoma patients with AA consistently occur later than MG, indicating that while there is a correlation in their activity, they are not fully aligned.

The pathogenesis of AA is not yet fully understood, but its typical histological characteristic involves the infiltration of inflammatory cells around hair follicles, particularly CD8^+^ T lymphocytes, which correlates positively with disease severity. In this study, we found that all thymoma patients with AA exhibited an inversion of the CD4^+^ T/CD8^+^ T lymphocyte ratio in their serum (i.e., a relative increase in CD8^+^ T lymphocytes in the serum), which was significantly higher than that observed in the thymoma patients without AA. Therefore, we hypothesize that the relative increase in CD8^+^ T lymphocytes within thymoma tissues may be a causative factor for the development of AA in thymoma patients. Although most thymoma tissues from patients without AA also exhibited an inversion of the CD4^+^ T/CD8^+^ T lymphocyte ratio, it cannot be ruled out that abnormal T lymphocytes in the serum are associated with thymoma. We propose that the pathogenesis of AA may involve abnormal autoimmune CD8^+^ T lymphocytes produced by thymomas bypassing the selection process in the thymic medulla and migrating to peripheral tissues [4,6,14], leading to the loss of immune privilege of hair follicles, the upregulation of inflammatory pathways, and autoimmune-mediated follicular destruction [8,9,15,16]. This may also explain why thymoma patients with AA often have other coexisting PNSs such as autoimmune myocarditis and dermatomyositis, which are mediated by cytotoxic T cells [9,17,18]. This is also observed in the difference in the pathogenesis of autoimmune diseases mediated by antibodies such as MG [19]. However, this study found that patients with AA had a significantly increased incidence of MG. Therefore, we hypothesize that abnormal CD8^+^ T lymphocytes produced by the thymoma can enhance the activity of CD4^+^ T lymphocytes, thereby promoting the production of autoantibodies and inducing the appearance of MG symptoms [20].

Our analysis also found that, while there was no difference in the pathological types of thymomas between patients with and without AA, the proportion of Stage IV tumors was significantly higher among thymoma patients with AA. Although most studies have shown that CD8^+^ T lymphocytes possess tumor-killing activity and that CD8^+^ T lymphocyte levels are positively correlated with tumor prognosis [21,22,23,24,25], this relationship may also depend on the immune status of the CD8^+^ T cells. If CD8^+^ T cells are in an “exhausted” state, their effector function becomes limited despite their high numbers, and an increased count may instead reflect poor tumor control [26,27]. Furthermore, whether the abnormal autoimmune CD8^+^ T lymphocytes generated in thymomas retain a normal tumor-killing capacity remains unclear [28,29]. Therefore, it is still uncertain whether the increase in the CD8^+^ T lymphocyte count is a cause or a consequence of proliferation and invasion of thymomas, and this question warrants further investigation through cytological studies. Our analysis also found that, while there was no difference in the pathological types of thymomas between patients with and without AA, the proportion of Stage IV tumors was significantly higher among thymoma patients with AA. Whether the increased number of CD8^+^ T lymphocytes promotes thymoma proliferation and invasion remains unclear and warrants further cytological investigation.

## 5. Conclusions

In summary, we hypothesize that the etiology of thymoma-associated AA may be attributed to abnormal autoimmune CD8^+^ T lymphocytes produced by the thymoma. Therefore, we have observed that treatments such as oral corticosteroids, intravenous immunoglobulin, and plasma exchange have shown limited efficacy for AA in thymoma patients in clinical practice [15,18,30]. Based on the results of this study and published case reports, surgical intervention remains the most effective means of improving thymoma-associated AA, which may be related to changes in T lymphocytes of peripheral blood following thymoma resection [16,31,32]. Due to the extremely low incidence of thymoma-associated AA, further analysis of its clinicopathological characteristics requires more clinical case data, and its pathogenesis warrants further investigation using cellular and animal models. Only by understanding the exact pathophysiological mechanisms can we deepen our knowledge of PNS and achieve precise prevention and treatment for these patients.

## Figures and Tables

**Table 1:** Clinicopathological and immunological characteristics of thymoma patients with alopecia areata (AA).

NO.	Sex	Age (Years)	WHO Histology	M-K Stage	MGFA Clinical Classification	Serum Antibodies		CD4^+^/CD8^+^ T Lymphocyte			AA Appeared Later Than MG (Months)	Combined PNS (Except MG)	AA Improved Later Than MG (Months)
						AchR Antibody	Titin Antibody	Serum	Tumor	Peritumoral Tissue			
1	M	40	B2	IVa	III	+	−	Inverted	Inverted	Normal	3	Dermatomyositis	3
2	M	41	B3	IVb	V	+	+	Inverted	Inverted	Normal	10	Leucoderma, Lichen planus	5
3	M	47	B2	IIIb	III	+	+	Inverted	Inverted	Normal	7	Hyperthyroidism	6
4	F	44	B2	IVa	V	+	+	Inverted	Inverted	Normal	51	Good’s syndrome, Interstitial pneumonia	5 (AA reappeared when the tumor recurred)
5	F	45	B2 mixed with B3	IVa	V	+	+	Inverted	Inverted	Normal	170	Good’s syndrome, Erythroderma	The tumor recurred and became alopecia total
6	F	46	B3	IVa	IV	+	+	Inverted	Inverted	Normal	12	Pure red cell aplasia	9 (AA reappeared when the tumor recurred)
7	F	53	B2 mixed with B3	IVa	I	+	+	Inverted	Inverted	Normal	6	No confirmed PNS	9
8	F	59	B2	IIIa	I	+	+	Inverted	Inverted	Normal	3	Limbic encephalopathy	3
9	F	72	B1	I	I	+	−	Inverted	Inverted	Normal	1	Autoimmune thyroiditis, Autoimmune myocardioptis, Sicca syndrome, Agranulocytosis	2

M, Male; F, Female; WHO, World Health Organization; M-K, Masaoka–Koga; MGFA, Myasthenia Gravis Foundation of America; MG, Myasthenia Gravis; and PNS, paraneoplastic syndrome; “+” indicates positive; “−” indicates negative.

**Table 2:** Comparison of clinicopathological and immunological features of thymoma patients with and without alopecia areata (AA).

Clinicopathological and Immunological Features	Thymoma Patients with AA (*n* = 9)	Thymoma Patients without AA (*n* = 45 or 30)	*p*-Value
Proportion of female	66.67% (6/9)	66.67% (30/45)	>0.999
Age [Median (IQR)]	46 (44, 53)	46 (43, 53)	0.889
B2/B3 type	88.89% (8/9)	55.56% (25/45)	0.075
IV Stage	66.67% (6/9)	8.89% (4/45)	0.001
Combined MG	100% (9/9)	66.67% (30/45)	0.049
Class IV-V of MGFA	44.44% (4/9)	20.00% (6/30) ^†^	0.197
Postoperative Myasthenic Crisis Occurrence	22.22% (2/9)	6.67% (1~2/30) ^†^	0.223
Combined PNSs other than MG	88.89% (8/9)	6.67% (3/45)	<0.001
AchR antibody (+)	100% (9/9)	84.44% (38/45)	0.586
Titin antibody (+)	77.79% (7/9)	71.11% (32/45)	>0.999
CD4^+^/CD8^+^ T lymphocytes was inverted in serum	100% (9/9)	24.44% (11/45)	<0.001
CD4^+^/CD8^+^ T lymphocytes was inverted in tumor	100% (9/9)	86.67% (39/45)	0.574

IQR, Interquartile Range; MG, Myasthenia Gravis; MGFA, Myasthenia Gravis Foundation of America; and PNS, paraneoplastic syndrome. ^†^ For the data on MG classification and the occurrence of postoperative myasthenic crisis, only thymoma patients with MG were included. Therefore, in the thymoma patients without AA group, the denominator was 30 rather than 45.

## Data Availability

The datasets used and analyzed during the current study are available from the corresponding author upon reasonable request.
